# Peer Support Workers in Health: A Qualitative Metasynthesis of Their Experiences

**DOI:** 10.1371/journal.pone.0141122

**Published:** 2015-10-30

**Authors:** Jennifer MacLellan, Julian Surey, Ibrahim Abubakar, Helen R. Stagg

**Affiliations:** University College London, Research Department of Infection and Population Health, 4^th^ floor Mortimer Market, off Capper Street, London, United Kingdom; Iranian Institute for Health Sciences Research, ACECR, ISLAMIC REPUBLIC OF IRAN

## Abstract

**Objective:**

Peer support models, where an individual has a specific illness or lifestyle experience and supports others experiencing similar challenges, have frequently been used in different fields of healthcare to successfully engage hard-to-reach groups. Despite recognition of their value, the impact of these roles on the peer has not been systematically assessed. By synthesising the qualitative literature we sought to review such an impact, providing a foundation for designing future clinical peer models.

**Methods:**

Systematic review and qualitative metasynthesis of studies found in Medline, CINAHL or Scopus documenting peer worker experiences.

**Results:**

1,528 papers were found, with 34 meeting the criteria of this study. Findings were synthesised to reveal core constructs of reframing identity through reciprocal relations and the therapeutic use of self, enhancing responsibility.

**Conclusions:**

The ability of the Peer Support Worker to actively engage with other marginalised or excluded individuals based on their unique insight into their own experience supports a therapeutic model of care based on appropriately sharing their story. Our findings have key implications for maximising the effectiveness of Peer Support Workers and in contributing their perspective to the development of a therapeutic model of care.

## Introduction

The use of peer support models in healthcare is well established in mental health services where peer support workers (PSWs) serve to improve engagement with healthcare and positive health outcomes among their clients [1–15]. PSWs are usually recruited from the same client pool as the individuals that they are looking after, thus sharing similar experiences or characteristics with the target intervention group. They are given a basic level of training or orientation to the role and join a team of PSWs to offer support and encouragement to others on their illness journey [16]. This can range from informal visits and sharing of experiences to formal appointments focused on practical information giving and support in relation to the intervention.

The ability of the PSW to engage with clients on the same level through an understanding of the challenges of their situation is a core feature of their effectiveness. Of further significance is the fluidity of the peer worker role, enabling them to move successfully between client and service provider roles to facilitate client wellbeing and positive care outcomes. Such outcomes could include attendance at hospital appointments, HIV medication adherence, self monitoring of blood sugar, commitment to a healthy diet and exercise regime, or continuation of breastfeeding. The value of peer support models for improving client access, uptake and engagement with health services and for adding value to the client experience is widely recognised. Their success has been replicated in engagement with hard to reach groups from middle aged men with chronic disease [17–21], to those with stigmatised disease [16,22–25] and people who misuse substances [26,27].

In spite of the number of studies qualitatively analysing the impact of the role on the PSWs in healthcare, their focus is often narrow and related to issues of personal recovery. The over-arching experiences of PSWs, as described in their own words, have not been systematically synthesised. Collating this evidence is a vital task to bring together what is known with the aim of maximising the effectiveness of PSWs. Furthermore, such synthesis plays a valuable role in contributing PSW perspectives into the design of standardised guidance for the planning and training of future PSW intervention programmes in healthcare. This review aimed to fill this evidence gap by systematically reviewing PSW perceptions of their experiences of the role from qualitative studies and synthesising findings to identify common constructs in these experiences in order to provide new understandings from the data. We also assessed how this evidence could impact PSW effectiveness and promote the standardisation and thus potential accreditation of peer support work.

## Methods

The meta-synthesis process aims to draw together qualitative studies from a related area and search for relationships across records to expose new perspectives and understandings of a phenomena. Systematic and reproducible search techniques are thus used to identify relevant records to the topic under study, quality assessment of records are made, findings are then presented and related to each other to gain new insight [28].

### Systematic Literature Search

This review investigated qualitative studies documenting PSW perceptions of their experiences of the role. The search terms utilised were (“peer supporter” or “peer mentor” or “peer educator” or “peer advocate” or “peer listener”) AND “health”, thus capturing the broad use of terminology for the PSW experience. These were informed by a wide selection of preparatory reading as no MeSH terms covered the topic. Experts in the area were consulted (HRS, IA) to ensure the use of appropriate and wide search terms. For consistency in this synthesis, the term PSW is used throughout.

### Inclusion and Exclusion Criteria

Our inclusion criteria were for articles to report PSW experiences of face to face peer support models as some component of the primary qualitative data. All health domains relating to adults were included, from all geographical locations. Records were excluded if they did not contain information on the experience of peer support work from the perspective of the peer in a health context. Online and telephone peer support models were excluded due to the lack of face-to-face interaction and the complicating dimension of online communication styles and rules of interaction. Records reporting client outcomes without inclusion of the PSW experience were also excluded. Peer work with adolescents in schools was excluded as a unique domain of practice with the peer usually recruited from the target rather than the affected population. Only publications available in English were reviewed.

### Search Results

Records were de-duplicated, then screened for inclusion by the two reviewers JM and JS, with a third reviewer available to resolve any discrepancies (HRS). Inter-rater agreement for screening of full text records was 95%.

### Literature Synthesis

JM and JS independently read the records and summarised the key constructs as interpreted by the research authors into an Excel grid to facilitate the cross-comparison process of the synthesis. The initial agreement level in coding was 87% between JM and JS. Meta-synthesis is influenced by the perspective of the researchers as they aim to inductively generate higher order constructs from the author themes of the records. This prompted extensive discussion and refinement of coding decisions before 100% consensus was reached.

Since there is no universally recognised method of synthesis, each record was listed alphabetically and constructs from each paper were compared with the next in the alphabetical list [29]. As the records were synthesised, categories were merged and collapsed while the researchers remained open to emergent themes. Synthesis of commonalities and contradictions across records built an explanatory model or ‘line of argument’ of the phenomenon, bringing fresh interpretation to the topic. This approach to meta-synthesis has its origins in grounded theory and aims to develop a model inductively from the data [30].

### Critical Appraisal

Study quality was graded using the Critical Appraisal Skills Programme tool for qualitative studies (CASP) [31]. The two reviewers (JM and JS) graded the records independently, conferred on a random selection and discussed discrepancies to achieve consensus, with the third reviewer available to resolve any discrepancies (HRS). Records were not excluded as a result of a low score, recommended by Atkins *et al*. [29], although they were integrated to this synthesis with these concerns in mind. Such records tended to be descriptive rather than interpretive, and contributed fewer insights to this synthesis.

Medline, CINAHL and Scopus databases were searched during June 2014. In order to identify potentially eligible studies not available through these databases, manual searching of article reference lists and grey literature supplemented the findings.

## Results

**Fig 1:**
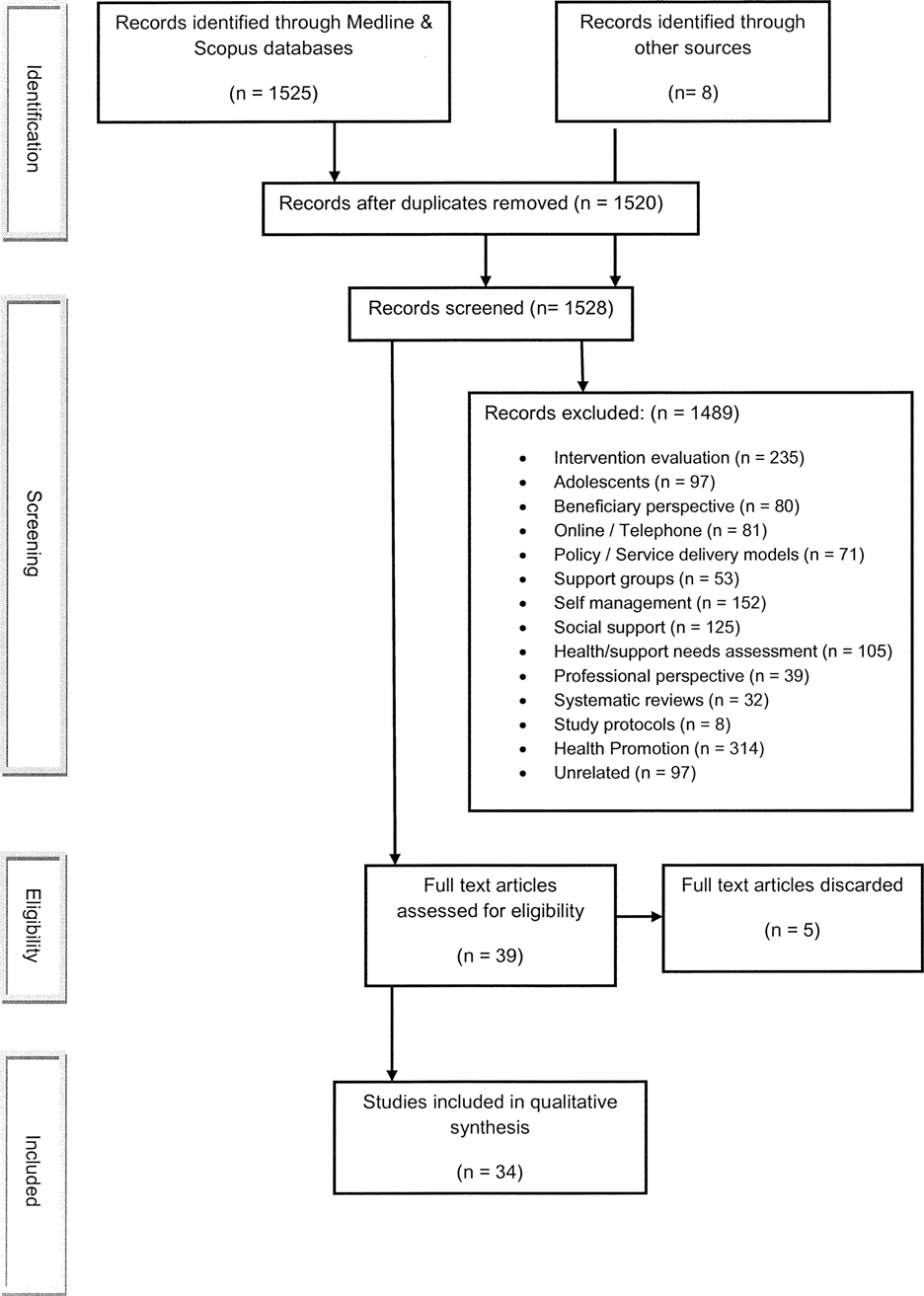
Search strategy for meta-synthesis of Peer Support Worker experiences of working as a Peer Support Worker in the Health Service context.

The three databases yielded 1,520 publications post deduplication, plus 8 records from manual searching of article reference lists and grey literature. Search Results are recorded in the PRISMA diagram (Fig 1). Eligibility criteria for this review was fulfilled by 39 articles. On full text analysis, a further 5 articles were discarded as not providing a peer perspective, leaving 34 records to complete the objectives of this paper.

Table 1 presents the 34 included records. Study participants ranged from 1 (study presenting a narrative) to 154 (completing an online questionnaire). All were self-selecting, although some efforts were made by the researchers to purposively sample to reflect participant diversity across project sites and in demographic characteristics. The principle method of data collection was in-depth interview (18) followed by focus group discussion (3), or a mix of both these approaches (5). One study presented a peer narrative without researcher interpretation while the remaining 7 included studies employing a combination of methods, such as questionnaire and observation.

**Table 1:** Summary of included records and principle constructs.

Study	Reference	Country	Discipline	Participants	Method	CASP	Themes
1	Aiken & Thomson 2013^42^	UK	Breastfeeding	19	Focus group & Interview	8	Use of self, Roles & Boundaries, Responsibility.
2	Aoun *et al*. 2012^17^	AUS	Weight reduction	15	Focus group	8	Responsibility, role modelling, connection.
3	Barg *et al* 2011^18^	USA	Heart Disease	10	Interview	8	Giving back, belonging, connection.
4	Barlow *et al* 2005^19^	UK	Chronic Disease	11	Interview	7	Contributing, belonging, confidence.
5	Bouchard *et al* 2010^1^	CA	Mental health	10	Interview	9	Safe, fulfilled, social bond, improved quality of life.
6	Brunier *et al* 2002^33^	CA	Renal disease	31	Interview 0,4,8,12 months	7	Developing lasting friendships, Learning from losing, Reciprocity, Contributing.
7	Croft *et al* 2013^25^	UK	Tuberculosis	6	Interview	6	Making sense of the past, Renewed self, Connectedness/belonging.
8	Curtis *et al* 2007^34^	UK	Breastfeeding	7	Focus group	7	Reciprocity, Constraints in working relationships.
9	Dennis 2002^35^	CA	Breastfeeding	30	Interview	7	Contributing, connection, enhanced self-esteem.
10	Dutcher *et al* 2011^16^	USA	HIV	23	Interview	8	Valued, role modelling, unique role.
11	Gillard *et al* 2013^2^	UK	Mental health	15	Interview	8	Giving back, reframing identity, boundaries.
12	Greenwood *et al* 2013^3^	UK	Mental health	4	Interview	7	Connection, giving back, regaining identity, development of skills.
13	Gusdal *et al* 2011^24^	Ethiopia & Uganda	HIV	17	Interview	8	Reframing identity, meaning from illness, unique position of role.
14	Hilfinger *et al* 2009^23^	USA	HIV	6	Interview	7	Sharing story, reciprocity, validation of their life.
15	Hutchinson & Lovell 2013^4^	UK	Mental health	6	Participatory Action Research	9	Connection, reciprocity, reframing illness narrative, participation.
16	Ingram 2013^53^	UK	Breastfeeding	7	Interview	7	Role clarity, gatekeeping of clients, role & advice challenges for midwives.
17	Kemp & Henderson 2012^5^	AUS	Mental health	7	Focus group	8	Role clarity, team perception, expectations, self disclosure.
18	Marino & Simoni 2008^22^	USA	HIV	16	Interview & Focus group	8	Social acceptance/belonging, reciprocal support, personal growth/reframing identity.
19	Moll *et al* 2009^6^	CA	Mental health	6	Interviews	7	Defining role, boundaries, identity transition, responsibility, belonging.
20	Moran *et al* 2012^7^	USA	Mental health	31	Interviewed twice	9	Restorying past, belonging, awareness of other.
21	Moran *et al* 2012b^8^	USA	Mental health	30	Interview & questionnaire	8	Giving back, connected, reciprocal network.
22	Mosack *et al* 2013^20^	USA	Hypertension	114	Survey, observation, focus group	7	Improved self care, responsibility.
23	Mowbray *et al* 1998^13^	USA	Mental health	11	Interview	6	Skill development, connection, value, boundaries
24	Murphy *et al* 2008^41^	UK	Breastfeeding	24	Interview & questionnaire	9	Disheartened if unable to make contact, frustration if no connection, sharing experience, time management.
25	Norman *et al* 2008^26^	AUS	Hepatitis C	1	Interview	5	Reframing identity, boundaries, evolving interpretation of life, professional support.
26	Paul *et al* 2013^21^	IRE	Diabetes	16	Focus group & interview	6	Valued professional support, unique role, want more updates.
27	Pillemer *et al* 1996^32^	USA	Alzheimers	45	Questionnaire, interviews, focus groups	7	Responsibility, reciprocity, unique position of peer, belonging.
28	Salzer *et al* 2013^9^	USA	Mental health	154	Online survey	7	Skill development, responsibility to self and others, increased confidence.
29	Scott & Doughty 2012^10^	NZ	Mental health	37	Focus groups	6	Peer support relational, documentation demands don't fit recovery model, collaborative notes.
30	Walker & Bryant 2013^11^	CA	Mental health	N/A	Metasummary	8	Restorying past, multiple identity, outsiders, transition challenging, boundaries.
31	Watson 2013^12^	UK	Mental health	1	Narrative	6	Restorying past, reclaiming identity, giving back, responsibility to self.
32	Weeks *et al* 2006^27^	USA	Substance misuse	99	Interviews & observation	6	Personal worth, connection, new identity, responsibility, self awareness.
33	Whittemore *et al* 2000^14^	USA	Mental health	10	Logs, Interviews, focus groups	9	Helping others, sharing experience, reciprocity, responsibility to self and others.
34	Yuen & Fossey 2003^15^	AUS	Mental health	3	Interviews	8	Connection with others, responsibility to self and others, reciprocity, role clarity.

AUS = Australia, CA = Canada, IRE = Ireland, NZ = New Zealand, UK = United Kingdom, USA = United States of America

¹ = Reference.

The field of mental health gave the greatest yield of records to meet the criteria of this review (14), followed by peer work in non-communicable diseases (9) ranging from diabetes and heart disease to weight management and renal failure. Infectious disease followed (6), focusing on HIV peer support workers, with hepatitis C and tuberculosis having a presence. Breastfeeding and new mother support added the final 5 articles.

The synthesis revealed two core constructs that provide insight and possible explanation of effectiveness in the PSW role. These were:

1. Reframing of identity through reciprocal relations
2. Therapeutic use of self, enhancing responsibility

**Fig 2:**
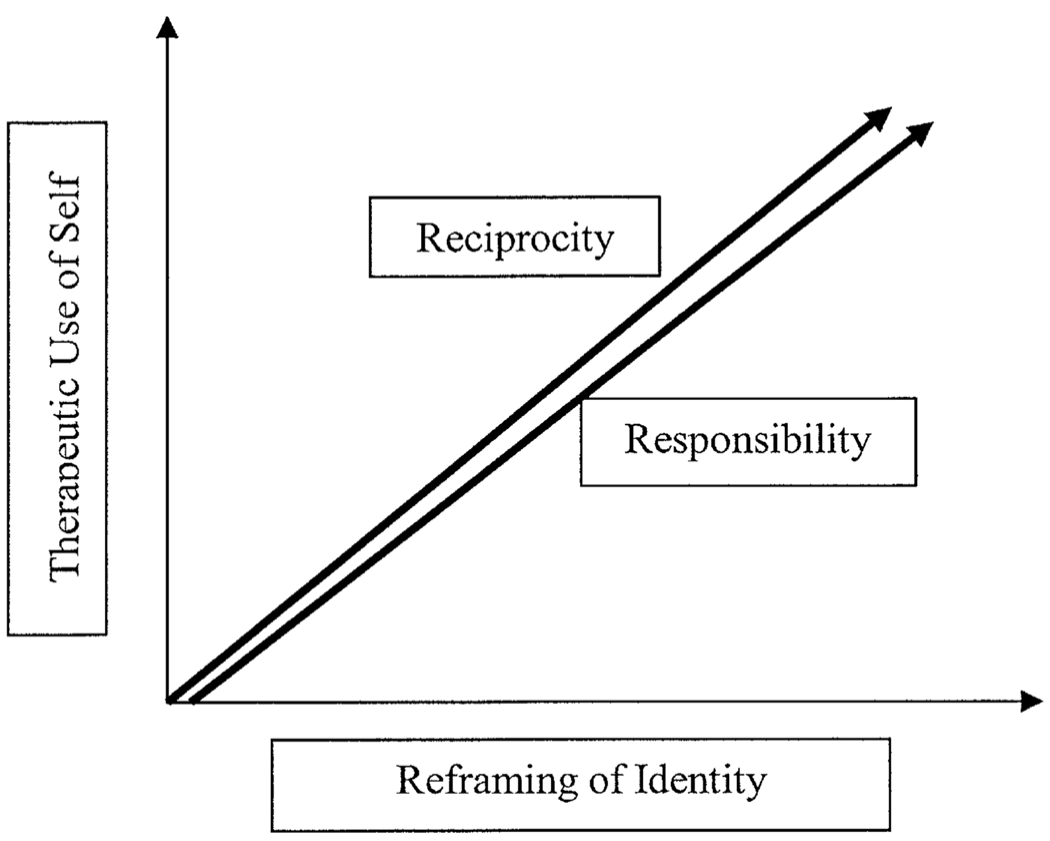
Metasynthesis constructs representing a conceptual framework of PSW effectiveness. As the therapeutic use of self increases in the PSW-client relationship, reciprocity increases. As the reframing of identity process progresses in the PSW, the sense of responsibility to the client, colleagues and self also increases.

These core constructs form the PSW conceptual framework emerging from this synthesis, and expose the complex and inter linked sociological phenomena of PSW experience. As can been seen in Fig 2, with an increased incidence of the therapeutic use of self care model by the PSW, there is a corresponding increase in both the sense of responsibility to others and the self, and in the extent and quality of the reciprocal relationship with the client and colleagues. This relationship is mirrored on the identity axis, as the PSW experiences an evolution and reframing in their identity. The quality and extent of reciprocal relations formed through their role are increased with a corresponding rise in the sense of responsibility to themselves and others. Naturally this theoretical framework is not necessarily linear in reality as individuals oscillate along the axes of their experience. However, we feel the linear representation aids conceptual understanding of the synthesised constructs. Each core construct is discussed in detail under strengths of PSW experiences and challenges in their experiences, with discordant findings across records and constructs reported in the challenges section.

### Reframing of identity through reciprocal relations

#### Strengths of PSW experiences

A consistent author construct across records was the benefit of the PSW engaging in a reciprocal relationship through their work. Analysis of this construct revealed interlinking aspects with reciprocal relations and the evolving identity of the PSW. Through interaction with other PSWs and clients, the benefits of belonging and reframing of the past to give meaning to their suffering and experiences, enhances an awareness of their ability to participate and give something back. The evolution of their identity into this giving role is stimulated by the relationships, and also develops and further benefits those relationships.

Reciprocity reflects a bi-directional relationship, stimulating a greater degree of personal insight and awareness of the impact of PSWs behaviour on others [1,8,14]. Such insight into the self led to greater self acceptance and understanding of their own position on the recovery journey [4,8,12,13,25,27]. Greater insight into others’ experiences put individual egos aside and led to an appreciation of what others had been through [3,8,13,19,25]. Close PSW teams were facilitated by their shared illness experience, shared programme goals and support of each other’s different positions in the recovery journey [3,4,12,15,22]. Connection with other PSWs was highly valued and allowed individuals to feel safe as themselves, integrating their psychiatric or physical condition into their life [1,3,4,8,22,32]. Furthermore, insight of the PSWs into their own personal illness led to regaining control over their life by actively participating in their illness management and recovery. The reciprocal nature of the PSW and client network, embodying social participation, was specifically linked to the peer’s recovery in the study by Moran [7], but was also seen in a mental health, infectious disease and non-communicable disease context [8,12,13,16,20,24,27]. While this insight helped to define the PSW identity, it increased their awareness of the presence of their overlapping identities [3,4].

Participation in social networks with clients and other PSWs acted as both a motivation and a support in reducing feelings of isolation in 14 of the articles in this synthesis [1,3,11,13,15,17,18,22,27,32–35]. Social participation has been shown to remove anxiety and unhappiness while the existence of social relations increases the motivation for helping [36]. Helping others in the role of PSW was felt to be a worthwhile and positive job [3,13,27]. Pillemer *et al*. reported 88% of their 45 respondents cited ‘helping others’ as their principle motivation for joining the PSW programme [32]. Altruism is described by Mollica as one of the most powerful social tools to stimulate an individual’s self-healing [37]. This motivation supports development of the PSW identity, by repaying the gift of having their story heard by colleagues and clients through telling the client’s story and making them visible. This motivation was reported across the papers of this review [1,2,14,22–24,27,32,33].

While combating personal isolation, in some cases participation also eased the pressure in the relationship with their partner [34], taught the PSW to ‘stay clean’ [27], and to receive acceptance and validation of their new recovery identity [3,4,6,12,27,38]. The easy acceptance of each other in the PSW teams produced a safe and positive work environment in the study by Mowbray *et al* [13]. In such an environment, the intangible ‘smoky’ stigma and fear surrounding their psychiatric illness was described as becoming positive, creating an air of recovery, and even assuring recovery for participating individuals [8]. Joining the PSW network was also described as facilitating reintegration into the community [11,19]. This was a result of doing something perceived as positive for the community, not just receiving but participating [27,33,35].

The belongingness theory of Baumeister and Leary describes this need to belong as a fundamental human motivation [36]. Part of a person’s identity and self-understanding is their place in relation to family, friends and their community. If these relations are severed or compromised, as in the case of many of the subjects presented in the studies of this review, it fundamentally impacts the person’s own self understanding [39]. This is based on the belief that individuals are socially embedded, where social relations constitute an individual’s identity and this is reaffirmed or discredited through interaction with others. Since the PSWs’ emerging identity is dependent on this interaction, it was seen to engender a sense of purpose and increased self esteem [9,11,12,19,21,22,25,33–35,40].

The reciprocal relations with others encouraged goal setting by the PSWs relating to what type of person they wish to be in the studies by Freeman *et al* and Moran *et al* [8,39]. Their life and illness experiences gained new meaning and involved not denying the past but validating and reframing it to accept who they are [1–3,8,12,14,22,23,32,33]. It gave meaning to suffering and made it all worthwhile [3,8,33]. Thus the PSW learns to reinterpret their own life story as a positive experience, worthwhile because of what they can now give to others. This reinterpretation or reframing of identity is an essential experience as it is not possible for individuals to be extracted from the context and histories within which they understand themselves. Acceptance of their illness or history in their current relational context reflects evolution of the self seen through personal validation and a positive outlook [39]. This was seen to translate into feeling more hopeful about the future, affirming their new PSW role identity and a general improvement in quality of life [1–3,9,12,14,15,18,19,22,23,25,27,32,33].

#### Challenges in PSW experiences

We examined author constructs in the records to expose discordant findings in our synthesised themes. Across the 34 records there was a significant variety of experience with contrasting findings supporting our constructs. For example, an emotional disconnect from the PSW was reported if they felt they were not needed. They described a lost opportunity if they were unable to connect with a client [14,17,21,35]. Those PSWs who did not experience positive engagement with clients often reacted with a lack of motivation to continue in the role and emphasised the time commitment required by the programme [41]. However, it was also acknowledged that the PSW intervention was perhaps not appropriately timed for some clients [22,23]. In Murphy *et al*. when clients were unaware of the role of the PSW there was frustration and difficulty in making a connection [41].

PSW work was described as relational and not captured in service documentation [10,23,42]. Most information was described by PSWs as being carried in their head, which increases trust from the client, but clashed with the note writing culture and accountability of the mental health system [10]. PSWs described how patient behaviour can be disciplined by the information kept about them in the medical notes. They reported writing collaborative notes in an empowering way, or struggling to manage time for the administrative commitment [5,10,42].

This first synthesised construct is intimately linked with the second: Therapeutic use of self enhancing responsibility. This is because the developing reciprocal relationship enhances the peer identity and sharing of one’s story is a unique component of this identity. Sharing one’s story and realising its impact through the relationship (therapeutic use of self) stimulates a sense of responsibility to the client, the programme, colleagues and to the self.

### Therapeutic use of self enhancing responsibility

#### Strengths of PSW experience

Analysis of the common author constructs of sharing one’s story and responsibility revealed a unique body of practice in the therapeutic use of self by the PSW as the core component of their effectiveness. The type and level of disclosure was related to a sense of responsibility to the client but also to the PSW themselves.

Some PSWs in this synthesis described their position as unique, drawing dignity and pride from a belief in their unique skills [43]. The sense of responsibility and commitment PSWs brought to their work can be seen as a way to import meaning into their role. This is an important source of dignity for workers whose role is perhaps undervalued or invisible in the health economy [43]. Focusing on their current recovery position and reframing the past through the lens of the present, to offer a model of recovery, was seen as a foundation of the PSW role [2]. In many of the records reviewed for this synthesis, the sharing of personal experiences was described as a unique characteristic of the PSW and a mechanism for gaining trust with clients [3,16,23,25,41].

Sharing could be supportive through the giving of advice and assistance with problem solving [1,3,14,22,23,26,33,41]. Clients could openly discuss with the PSW their drug use and their feelings of having let the medical staff down by having a relapse [26]. Judgement of client behaviour, whether perceived or real, was described as a huge barrier to engagement and the PSW felt they could alleviate this [16,26]. Their equality of relationship put the PSW in a unique position to help as they could move between the role of service user and service provider depending on the requirements of the situation [21,23,26,32,44]. In both HIV and hepatitis C treatment contexts, PSWs described their role as integral because the treatment process is so complex and challenging due to multidisciplinary involvement and issues of stigma among the client group [16,26].

Sharing personal experiences is an important component of the recovery journey as individuals search for coherence in their challenging experience of illness or marginalisation [45]. Most people who have faced adversity are interested in how their story can help others. While teaching others about survival, they are also transferring some of their suffering to the listener and extending their own healing process [46]. The self-disclosure of the PSW also acts as validation of who the PSW is and what they have survived to tell [47]. Having a listener for your story is part of the therapeutic process of the reciprocal relationship, both recognising and supporting the storytellers’ healing process [48]. The PSW shares their story and in exchange the client discloses their experience. Self-disclosure from the PSW as ‘service provider’ is known to promote client engagement, rapport and trust in a therapist-client context [49]. This style of engagement models the therapeutic use of self, a powerful but unmeasurable technique used in the establishment of a positive therapeutic alliance in psychosocial care contexts and vital to the achievement of positive outcomes [50].

Part of this therapeutic contract is the normalising of the client experience and creation of an egalitarian relationship [49]. The PSWs of this synthesis felt they could achieve this through their ability to enjoy group membership in both service user and service provider roles. Being able to understand the clients’ frame of reference, or practising empathy, has been shown to link to positive outcomes and form the foundation of a positive therapeutic alliance [51,52]. Being caring, asking the client questions, discussing options and providing explanations are all positively associated with the therapeutic alliance, satisfying the basic needs in the client of autonomy and belonging. Giving directions and advice were negatively associated with a positive relationship [52].

Working as a PSW gave a sense of responsibility to the peers to the extent that one PSW described joining the programme as a ‘moral obligation’ [8,14,17,25,27,32]. Some PSWs approached the position with a dedicated work ethic and desire to be successful [14,18]. In some cases this led to increased motivation for self care behaviour and illness management to live up to the perceived expectations of service users and act as a role model [1,8,17,21]. In one study this led to a reduction in the use of acute services by the PSWs [9]. Even if the PSW described having a ‘relapse’, they felt encouraged to return to the PSW programme through this sense of responsibility to the clients and fellow PSW colleagues [12,13].

Some participants saw the PSW role as a stepping stone back into employment with the benefits of gaining skills, having money, and practicing scheduling [6,11,13,15,25]. The structure and responsibility involved with going to a job every day and focusing energy on something deemed as constructive work was described as strong treatment [8]. Learning communication skills that encouraged assertiveness rather than aggression and being able to talk to medical staff and clients without offending them were seen as valuable lessons [13,22,34]. This helped PSWs feel more professional in their conduct [2]. It also gave an opportunity for the development of transferable, under-developed skills and increased personal flexibility [3,33,34,44].

The work environment played a significant role in the experience of the PSW. In a positive environment, staff were described as treating PSWs as equals. Peer experiences were respected, they felt treated as a whole person rather than as their diagnosis, and strengths were emphasised [6,8,15,25]. Role playing different ways in which to respond to situations where boundaries are challenged was felt to be particularly valuable in the mental health PSW study reported by Gillard *et al*. [2].

#### Challenges in PSW experience

The discordant findings linked to this core construct supported the synthesis findings, for example if the PSWs were unable to share their story and work according to a therapeutic model of practice, support and assistance in the drawing of boundaries was requested. Generally, transition from the role of service user to PSW was described as challenging, particularly as to where to draw the line between service provider and friend [2,5,6,11,13,19,23,26]. It was described as a hard place, occupying the middle ground between provider and service user [2]. However, more engagement from clients was reported when the PSWs socialised with them and the relationship became more reciprocal [13,14]. Managing the power imbalance between client and health provider through transition to the role of PSW revealed some hostility against the system, and a ‘chip on the shoulder’ from the PSW side that had to be addressed [6]. Communicating with staff in a more professional manner was felt necessary to aid this role transition [6]. However, this required assertiveness and could be a struggle for some [2]. One PSW described the transition as getting to know the teachers and hoping you won’t get caught misbehaving [12]. The ‘best behaviour’ metaphor was also used by the PSWs in Gillard *et al* when referring to the pressure to gain acceptance from the team and the worry that if they get upset, people will think ‘she is having a service user moment’ [2]. This statement reflects the multiple, shifting identities of the PSW and their vulnerability.

The ‘lay expert’ position sometimes caused role confusion, with a golden rule stated by Watson that the PSW is never the expert [12]. Some PSWs reported feeling unsure of how to do the job or what was expected of them [6,12,13,15,26]. This lack of clarity was felt by some PSWs to be empowering [6,17], while others felt daunted [12]. Consequently, clarification in the extent and nature of PSW involvement at recruitment was deemed important [2,4,5]. This included knowing when to disclose their own story and how much to disclose. It was described as a fine balance, between sharing experiences and self preservation [3,5,6,12]. It also made the PSW vulnerable as their recovery journey is exposed to criticism by others [2,12]. Furthermore, care was necessary not to devote too much time to the PSW disclosure as this could detract clients from a willingness to engage [49]. Consequently, this drawing of boundaries was described as necessary to enact personal responsibility to the self [2,12]. The PSWs in Kemp *et al*. [5] recommended the provision of training in boundaries and ethics to overcome this danger, allowing them to manage the client relationship while keeping themselves ‘safe’. Interestingly, in Gillard *et al*.’s mental health study, PSWs reported non-peer staff to not value the ‘giving of personal experience’ as it crossed boundaries that were in place to protect the PSWs and service users. This tension was felt to constrain the emergence of a distinctive practice of PSW that the whole team could agree upon.

When feeling unsupported, some PSWs felt their role carried too much responsibility [13] or too much pressure to be a role model [6]. They felt inhibited about the extent to which they could share their experiences [2], and described feeling ‘out of their depth’ [6,26]. As a result, accessibility of a support person from this environment, either professional [5,13], in other PSWs [19,35] or from an outside network when there was no supportive PSW team [6,26], was mentioned as essential for managing some situations that arose [14,18,21,41]. However, professional supervision was not always felt to be constructive [2].

Finally, working with colleagues of different abilities caused frustration in the study reported by Yuen & Fossey as some could not do much without having a symptom relapse while others were ready to move on and be more active [15]. Thus clearly defined tasks would have been appreciated to fit the different abilities and motivation of the PSWs. Equally, being dependant on colleagues to facilitate a group that is then cancelled because they are on leave results in no payment and a feeling of powerlessness [2]. Regular updates of developments in treatments and advances relevant to the role were requested by some PSWs so they could give the most up to date advice [21]. Others requested more detailed medical information about the client to better target their advice [18].

Some discrimination and prejudice was experienced in the work environment with condescending humour from staff about clients in the mental health service [5,11]. In a hepatitis C context mistrust from clinicians was expressed through complaints about PSW behaviour [26]. This was also experienced among breastfeeding PSWs when the midwives would not refer clients to them for support [42]. Fitting in or being accepted in the workplace was a challenge for some [2,6]. A lack of identity and understanding of the PSW role by professionals was cited as a barrier and led to feelings of being an outsider [2,42,53]. With time, the breastfeeding PSWs interviewed by Ingram were ‘trusted’ with a wider range of mothers, but midwives found the role conflict and different advice given by the PSWs as a challenge they had to address [53]. This gatekeeping of access to clients and control over PSW activities was felt to constrain the effectiveness of the PSW role [2,34]. This was perceived by the PSW as the health professional being territorial, feeling threatened and being unwilling to relinquish ‘power’ [34]. This could relate to the practices of the PSW not corresponding neatly to existing clinical practice boundaries [2]. For example, by contributing practical suggestions in the team meeting but feeling a more psychodynamic approach is expected [2].

Balancing the volunteer PSW role and expectations of clients and health professionals with their own commitments was also described as challenging [5,26,41]. Others felt a sense of loss at the end of the PSW programme, or for clients who had died [22,23].

## Discussion

Systematically reviewing the literature on the experience of working as a PSW across 34 identified studies from a range of health disciplines has revealed diverse peer practice models applied across health contexts. However, regardless of whether the PSW is working in renal care, substance misuse, or with carers of relatives with Alzheimer’s disease, new insights relating to the reframing of identity through reciprocal relations and the therapeutic use of self, enhancing responsibility have emerged and appear shared across these contexts. These insights form the core constructs of the PSW conceptual framework emerging from this synthesis, exposing the complex and inter linked sociological phenomena involved in the PSW experience.

Traditional boundaries of provider-client separation originate in the medical model of clinical care and aim to guard against professional misconduct while preventing unhealthy dependence and emotional attachment [54]. However, contemporary post-structural therapeutic approaches in social work care advocate transparency and a deconstruction of power relations between professional and client. The model proposed by O’Leary *et al* [54] moves away from separation to a promotion of connection and the use of self. Boundaries or limits of the relationship are agreed between the client and social worker. Flexible arrangements could include contact outside of working hours, disclosure of personal details of the worker where it is relevant to the aim of the relationship, and the sharing of food or drinks [54]. These activities directly reflect the core components of the PSW role, and could provide theoretical direction to an accredited PSW model of practice.

### Limitations

The principle limitation of this synthesis is the reliance on other authors’ interpretations of their findings and selection of evidence in opposition to direct reporting from PSWs of their experiences. Challenges were carefully reported in the literature to perhaps avoid any blaming. This leaves the recommendations for components of a standardised peer model arising from this review more focused on positive aspects than situations to be avoided. Consequently, any peer support model influenced by this work would require ongoing assessment and potential revision.

Due to time constraints, coding and categorising of all the studies of this synthesis was completed by JM. A random selection of these studies was then read, discussed and coding revised accordingly with JS. The agreement level in coding was 87% between JM and JS, which prompted a review of an additional 6 randomly, selected included papers to clarify the coding decisions, at which 100% consensus was reached. Although the fact that a selection, rather than all, studies were coded independently by both researchers could compromise the validity of the findings, this is unlikely because of the level of agreement reached in coding discussions and achievement of saturation with the additional 6 studies reviewed together.

The records reviewed for this synthesis reported different models of peer support with varying degrees of PSW preparation, PSW–client contact, formal and informal contact structures, different contact environments and frequency of contacts. Often the details of the model design were described very briefly. All of these factors impact on the outcome and experiences of the PSWs and would inform recommendations to increase effectiveness of the PSW role. Since this information was not routinely available in an appropriate level of detail, structural programme recommendations are not available from this synthesis. It is recommended that a ‘realist review’ is conducted of published model designs to achieve this aim.

PSWs were represented from a variety of health domains though there was no specific analysis of the experiences of older people or ethnic minority groups. Thus it is not reliable to relate the findings of this synthesis specifically to different social populations, however there were older people included in the Alzheimer’s PSW study of Pillemer *et al* [32] and black American participants in the HIV PSW study by Hilfinger *et al* [23].

Furthermore, the restricted geographical distribution of included research reflects the focus of inquiry on the peer experience. This is in contrast to the outcome evaluations and reports of beneficiary experiences of peer support that proliferate across multinational contexts.

### Implications

The greater understanding of benefits and challenges of PSW experiences and their effectiveness from this synthesis calls for the greater availability of current peer support model details in the public domain. These practice models can then be joined with research findings to inform development of an accredited training course as recommended by Kemp *et al* [5]. Formalisation of the PSW role will clearly benefit PSWs, clients and health providers by informing expectations, facilitating positive working relationships and standardising remuneration for PSW time and expertise [24].

Acknowledgment of the emerging identity of the PSW using the theory of belongingness and its link to relationship formation and participation exposes positive psychological and lifestyle outcomes in the PSW. Furthermore, the ability of the PSW to actively engage with other marginalised individuals based on their own experiences emphasises the value of a therapeutic model of care based on the therapeutic use of self and the benefits of increasing responsibility to the self and others. This equalising of the client-provider power differential offers an exciting direction for practise and lies at the core of PSW effectiveness from this synthesis. Further exploration of PSW’s therapeutic use of self could inform formal design of an accredited model of peer practise, removing ambiguity from the role, standardising training, supervision and expectations. The benefits of the role to the PSW are a significant element of PSW interventions but decisions on their use should always be balanced with evidence relating to client benefit from the intervention.

## Conclusion

This synthesis of the literature exploring the PSW experience of their work has revealed useful insights into their effectiveness through analysis of benefits and challenges of their role. This exposes the PSW perspective for use in the future design of PSW models of effective practice. The synthesised constructs from this analysis identified the therapeutic use of self and reframing of peer identity as core to PSW effectiveness. These are influenced by the reciprocity of formed relations and the sense of responsibility of the PSW to clients, colleagues and themselves. The movement to a position of belonging and giving through the PSW programme supports the transition from social marginalisation to active participation in the role of PSW. The ability of the PSW to actively engage with other marginalised or excluded individuals based on their unique insight into their own experience supports a therapeutic model of care based on appropriately sharing their story. This offers an exciting direction for practise that equalises the power differential and separation ethos of traditional professional boundaries within the health service. Further exploration of this therapeutic use of self could inform formal design of an accredited model of peer practise, removing ambiguity from the role, standardising training, supervision and expectations.

## Supporting Information

S1 PRISMA Checklist (DOC)
